# Real-world experience with gene therapy in Duchenne muscular dystrophy center readiness and patients safety: report from Qatar

**DOI:** 10.1038/s41434-025-00580-3

**Published:** 2025-11-27

**Authors:** Mahmoud Fawzi Osman, Khalid Ibrahim, Claire Gleeson, Haytham Ibrahim, Ikram Ul Haque, Noora Alhamad, Tawfeg Ben-Omran

**Affiliations:** 1 Department of Pediatric Medicine, Sidra Medicine, Doha, Qatar; 2 Weill Cornell Medical College, Doha, Qatar; 3 Department of Physical Therapy, Sidra Medicine, Doha, Qatar; 4 Clinical Pharmacy service, Department of Pharmacy, Sidra Medicine, Doha, Qatar

**Keywords:** Gene regulation, Targeted gene repair, Biomarkers

## Abstract

Duchenne Muscular Dystrophy is a rare, X-linked neuromuscular disorder that leads to progressive muscle degeneration, loss of ambulation, and premature mortality due to respiratory and cardiac failure. Historically, Duchennke Muscular Dystrophy has been managed through supportive and symptomatic treatments, with limited options for disease modification. However, advancements in gene therapy have introduced promising interventions aimed at addressing the underlying dystrophin deficiency. Delandistrogene moxeparvovec (Elevidys) received accelerated approval from the U.S. Food and Drug Administration in June 2023 for ambulatory children aged 4–5 years with a confirmed diagnosis of Duchenne Muscular Dystrophy. This approval represented an advancement, offering a disease-modifying therapy at an early stage when muscle function remains relatively preserved. The Food and Drug Administration expanded its approval in June 2024 to include both ambulatory and non-ambulatory children aged 4 years and older. This study provides a retrospective real-world analysis of eight Duchenne Muscular Dystrophy patients who received Elevidys gene therapy at our center in Qatar. Recognizing the complexities involved in treating older Duchenne Muscular Dystrophy patients, a standardized protocol for pre- and post-infusion care was implemented. Our findings highlight the positive clinical outcomes of gene therapy for Duchenne Muscular Dystrophy patients in Qatar.

## Introduction

Duchenne Muscular Dystrophy (DMD) is a severe X-linked recessive neuromuscular disorder characterized by progressive muscle degeneration, leading to loss of ambulation, respiratory and cardiac complications, and premature death [1]. The disease is caused by mutations in the DMD gene, located on the Xp21 region, which encodes dystrophin, a crucial protein for muscle integrity [2]. DMD affects ~1 in 3500–5000 live male births worldwide [2].

The early symptoms of DMD typically manifest between 2 and 3 years of age, presenting as delayed motor milestones, difficulty climbing stairs, and a waddling gait [2]. As the disease progresses, most patients require wheelchair assistance by 10–12 years of age and develop respiratory insufficiency, often necessitating ventilatory support by their early twenties [3]. The primary cause of mortality in DMD patients is cardiac or respiratory failure, usually occurring between 20 and 40 years of age [3].

Genetic testing is the definitive diagnostic tool for DMD, typically performed following the detection of elevated serum creatine kinase (CK) levels [4]. Mutations in the DMD gene vary widely, with the most common being large deletions (~ 60%), duplications (~5–15%), and small point mutations, deletions, or insertions (~30%) [5]. These mutations disrupt dystrophin production, leading to the absence of a functional Dp427m muscle isoform, which is essential for maintaining muscle cell stability [5].

Current standards of care for DMD include corticosteroids and physical therapy to delay disease progression and manage symptoms [6]. Despite these interventions, there remains a critical need for disease-modifying therapies that address the underlying genetic cause of DMD.

Delandistrogene Moxeparvovec (Elevidys) is an adeno-associated virus vector-based gene therapy that is designed to deliver a gene that enables micro-dystrophin protein production, thus targeting the root cause of DMD [7]. It was first approved in the United States in 2023 for ambulatory children aged 4–5 years and later expanded to older ambulatory and non-ambulatory patients (aged 6 years and above) in 2024 (7, 8 & 10). The therapy has also been approved for use in Qatar and the United Arab Emirates [7].

In this retrospective case series, we present the Qatari experience of using Delandistrogene Moxeparvovec (Elevidys) in patients with confirmed DMD, focusing on treatment outcomes, safety, and clinical implications.

## Methods

### Data collection and patient selection

This study is a retrospective cohort analysis conducted through the review of electronic medical records (EMR) at Sidra Medicine. All patients with a confirmed diagnosis of Duchenne Muscular Dystrophy (DMD) were screened to identify those who met eligibility criteria for gene therapy.

Patients were considered eligible if they fulfilled the following inclusion criteria, in line with FDA regulatory guidance for Delandistrogene Moxeparvovec (Elevidys) administration:

- Age ≥ 4 years at time of treatment.
- Genetically confirmed diagnosis of DMD.
- Negative for pre-existing neutralizing antibodies against AAVrh74.

Exclusion criteria:

- Patients with any deletion in exon 8 and/or exon 9 in the DMD gene as per FDA criteria.
- We added two other exclusion criteria for safety reason:
  - Moderate or severe cardiomyopathy (ejection fraction <40% on echocardiography or clinical heart failure).
  - Ventilatory support (requirement for continuous non-invasive or invasive ventilation).

For patients meeting eligibility, detailed clinical, biochemical, and imaging data were collected. These included demographics, family history, age at presentation and diagnosis, clinical assessment including ambulation status, prior and ongoing use of corticosteroids or other therapies, respiratory support, cardiac involvement, laboratory assessments (serum creatine kinase [CK], alanine transaminase [ALT], aspartate transaminase [AST], troponin I, gamma-glutamyl transferase [GGT]), and cardiology evaluations (electrocardiography and echocardiography) before and after treatment.

### Pre-infusion protocol and patient preparation

Before treatment, the legal guardians of all patients signed an informed consent after a detailed discussion of the potential benefits, risks, and alternative treatment options for gene therapy. This consent process ensured that families were fully aware of the nature of the therapy, the pre- and post-infusion requirements, and the importance of regular follow-up.

In our practice we use the regular maintenance prednisolone dose of 0.75 mg per kg per day, which was reduced in some patients to 0.6 mg per kg per day as they did develop significant side effects. The dose was then increased to 2 mg per kg per day just two days before the infusions; this dose was continued and titrated during the first three months post infusions.

#### Antibody screening and infection control

All eligible patients were screened for antibodies against AAVrh74, the viral vector used in this gene therapy. Additionally, we confirmed that all patients were free of infections prior to infusion to further minimize immune-related complications.

Families were thoroughly educated about potential side effects of gene therapy, including Jaundice (hyperbilirubinemia), HyperCKemia (elevated CK levels pot-infusion) and changes in urine color (dark or reddish discoloration). Families were provided with our emergency contact numbers to report any concerns, ensuring rapid response and appropriate medical management if needed.

#### Infusion safety measures

To prevent potential immune reactions and infusion-related side effects, patients received pre-medications before gene therapy infusion IV methylprednisolone (1 mg/kg), diphenhydramine (1 mg/kg), and ondansetron (4 mg). Two peripheral IV lines were inserted to ensure safe and continuous administration without interruptions.

#### Intensive care and monitoring

The infusions were given in the pediatric intensive care unit (PICU), where patients were closely observed for any immediate reactions. The stay was 24 h in the PICU, followed by a 48-h stay in the pediatric ward. After discharge from the hospital, all patients attended weekly outpatient clinic visits for follow-up and continued physiotherapy three times per week.

#### Post-infusion monitoring

Patients were closely monitored for side effects and clinical improvement. Laboratory results, including serum CK, ALT, AST, Troponin I levels and Complete Blood Count were recorded before and after the infusion. Biomarker levels were monitored weekly for the first 12 weeks and then every 2–3 weeks.

#### Cardiac evaluation

All patients were assessed by an expert cardiologist specialized in the evaluation and management of muscular dystrophies, within 30 days prior to and after their gene therapy treatment. The assessment included a detailed medical history, physical examination, and Troponin I measurements conducted before treatment and weekly to every 2–3 weeks up to 30 weeks post-infusion. Additionally, each patient underwent an electrocardiogram and echocardiographic evaluation. Comprehensive echocardiograms were performed using commercial instruments from Philips Medical Systems (Bothell, Washington). Measurements taken included heart dimensions; left ventricular ejection fraction (LVEF), valve morphology, and tissue Doppler imaging of mitral and tricuspid annular velocities. Heart dimensions were measured using the parasternal short-axis view, and LVEF was calculated following Simpson’s rule.

## Results

In this study, we monitored the levels of various biomarkers (AST, ALT, CK, Troponin I, and GGT) in eight patients who received gene therapy for Duchenne Muscular Dystrophy (DMD) over a 30-week follow-up period. Patient demographics and their clinically significant characteristics are summarized in Table 1.

**Table 1:** Genetic and demographic data of all DMD patients enrolled in this study.

Patient	DOB	Age (years)	Gender	Nationality	Genetic result	Date of gene therapy infusion
1	22/07/2019	5	M	Lebanese	Deletion 45–52	25/06/2024
2	19/05/2016	8	M	Qatari	Deletion 45–47	17/09/2024
3	29/10/2013	11	M	Qatari	Mutation exon 19	24/09/2024
4	11/08/2020	4	M	Saudi	Deletions exons of 60 and 62	17/09/2024
5	13/07/2014	10	M	Qatari	Duplication exon 61, 62	27/08/2024
6	16/08/2015	9	M	Qatari	Deletion exon 18 to 48	27/08/2024
7	02/07/2019	5	M	Kuwaiti	Deletion exons 10 to 12	27/08/2024
8	08/09/2016	8	M	Qatari	Deletion exon 55	10/12/2024

The patients have shown documented improvements, particularly in biomarker levels and Timed Efficacy Assessments.

Serum CK levels decreased by an average of 44% within two weeks post-therapy and stabilized thereafter. ALT and AST levels showed initial reductions with some fluctuations, especially in older patients. Patient baseline and post infusion blood work are summarized in Supplementary Tables 1–8.

ALT and AST levels showed a decrease in the first week, followed by slight fluctuations and a minor increase around Weeks 2–3, after which a stabilization trend was observed from Week 4 onward. From Week 14, younger patients exhibited a gradual rise, peaking close to week 26 before returning to the initial post-infusion levels achieved around the second week. In contrast, older children experienced a steady decline in ALT over time, suggesting that younger children may have a longer adjustment phase following therapy (Supplementary Tables 1, 2).

During the follow-ups, CK demonstrated the most significant initial response, showing a sharp decrease of 44.1% in the first week. Levels continued to decline, reaching their lowest point around Week 4. However, CK levels significantly increased by 84.2% by Week 7. This pattern suggests a strong initial response to therapy, potentially reflecting muscle repair or reduced muscle damage, followed by a fluctuating phase as the body adapts (Supplementary Table 3).

GGT levels showed individual variability among patients, with Patient 2 and Patient 5 experiencing a moderate increase across the weeks, while Patient 3 displayed a delayed but significant increase in GGT levels by Week 14 that subsequently declined; other patients showed moderate increases that tended to stabilize around Weeks 4–5, suggesting an individualized response in GGT levels, with some patients demonstrating early adaptation and others a delayed response (Supplementary Table 4).

Cardiac evaluations included electrocardiograms and echocardiographic parameters, such as left ventricular ejection fraction and tissue Doppler imaging. Almost all patients had normal baseline cardiac evaluations, with the exception of one who had a slightly elevated baseline Troponin I level. After receiving gene therapy, none of the patients showed signs of myocarditis. This was confirmed by the absence of cardiac symptoms (such as chest pain, palpitations, or shortness of breath), normal cardiac examinations, and no significant changes in electrocardiograms.

Troponin I levels did not show significant rise for patients who had normal baseline levels, with the exception of Patient 3 that already had an above normal Troponin I level prior to infusion (51 ng/L, Troponin I: 0–20 ng/L) but remained consistent throughout the study, Supplementary Table 5 and Supplementary Fig. 1. Additionally, cardiac dimensions, left ventricular ejection fraction (LVEF), valve morphology, and tissue Doppler imaging of mitral and tricuspid annular velocities all remained within normal limits.

### Biomarker monitoring by age group

**Fig. 1:**
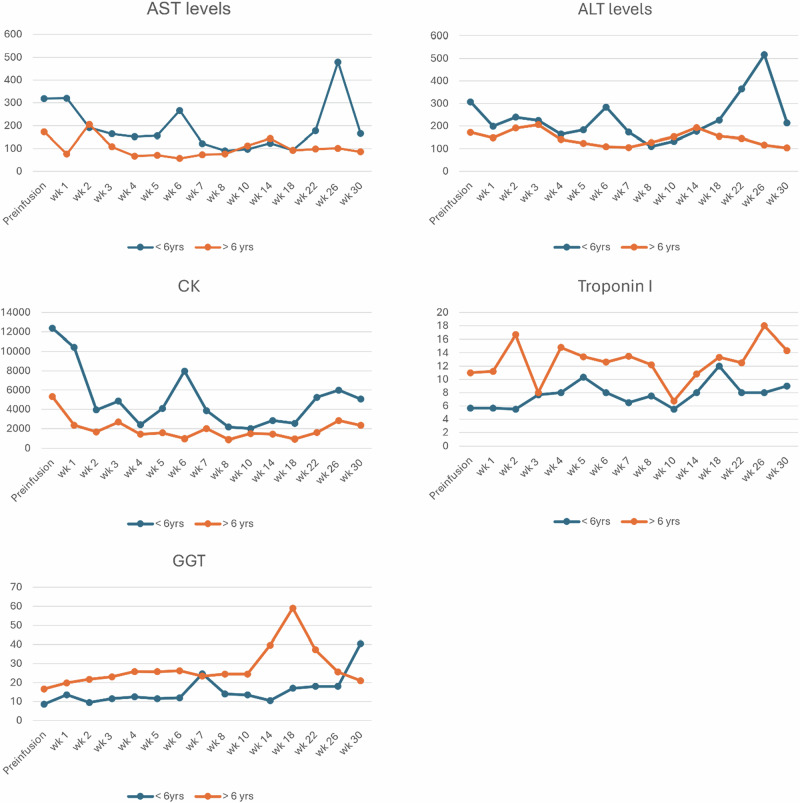
Biomarker change over 30-week follow-up by age group.

The comparison of biomarkers between patients older than 6 years and those 6 years or below reveals distinct patterns (Fig. 1).

Both age groups show a similar pattern with a slight decrease in ALT and AST levels post-infusion. However, younger patients tend to have marginally higher ALT levels over the weeks, particularly prominent in Week 24–26. Older patients display greater stability in their ALT levels. GGT levels are consistently higher in the older cohort, showing a steady increase over the weeks, with a pronounced spike by Week 7 and week 14. Younger patients demonstrate a more stable hepatic response, with a less marked increase in GGT levels.

Younger patients presented markedly elevated pre-infusion CK levels, which decline substantially by Week 2 but continue to fluctuate with noticeable peaks at Weeks 5, 7 and 26. In contrast, older patients have lower initial CK levels, following a similar declining trend but with less variability. Troponin I levels remain relatively low and stable in patients aged 6 years or below, indicating minimal cardiac stress. In older patients, Troponin I levels are relatively higher with observed peaks at Weeks 1 and 3, suggesting age-related differences in cardiac response to therapy.

### Timed efficacy assessments

The North Star Ambulatory Assessment (NSAA) Scores, The 10 Metre Walk Test (10MWT) and Get up time assessments were done for all patients before gene therapy and then one month and two months after gene therapy – Table 2. Two of the eight patients are non-ambulatory, making the assessments an invalid outcome measure for this group of DMD patients. Additionally, two patients from our cohort had returned to their home country after gene therapy, limiting our follow-up data. Among the remaining four patients whose scores and time were evaluated, two demonstrated improvements, one remained unchanged, and one experienced a decline.

**Table 2:** North Star Ambulatory Assessment, 10 Meter Walk Test and Get Up time of patients before and after gene therapy.

Patient	NSAA: Pre	10 MWT: Pre (m/sec)	Get up: Pre (sec)	NSAA: 1 month	10 MWT: 1 month (m/sec)	Get up: 1 month (sec)	NSAA: 2 months	10 MWT: 2 months (m/sec)	Get up: 2 months (sec)	Additional Notes
1	17/34	1.66	5	27/34	1.66	5	29/34	1.66	5	NSAA improved
2	10/34	0.99	21.16^a^	10/34	1.18	20.46^a^	10/34	1.2	20.22^a^	NSAA unchanged, functional improvement
3	0/34	DNA^b^	DNA^b^	DNA^b^	DNA^b^	DNA^b^	DNA^b^	DNA^b^	DNA^b^	Non ambulatory – NSAA not valid.
4	16/34	DNA^b^	DNA^b^	DNA^b^	DNA^b^	DNA^b^	DNA^b^	DNA^b^	DNA^b^	Did not attend follow-up appointment
5	2/34	–	–	2/34	–	–	2/34	–	–	Non ambulatory – NSAA not valid
6	30/34	3.2	2.49	31/34	2.97	3.27	33/34	3.02	3.41	NSAA improved
7	30/34	N/A	N/A	N/A	N/A	N/A	N/A	N/A	N/A	International patient - travelled
8	22/34	1.45	5.44	21/34	1.24	6.22	20/34	1.14	10.53	NSAA declined

^b^Did not attend.

^a^Using external support.

### Complications and other adverse effects

No severe adverse effects were reported. The most common side effect observed was corticosteroid-induced weight gain, which was managed with dietary adjustments and monitoring.

## Discussion

This study has described eight DMD patients, who successfully have received Delandistrogene Moxeparvovec (Elevidys) in a specialized center in Qatar.

This study explored the physiological responses to gene therapy in a cohort of DMD patients, assessing the impact on biomarker trends over a 30-week period post-infusion. All patients received comprehensive pre-infusion assessment including antibody screening, corticosteroids dosing, antihistamines, antiemetics, and supportive physiotherapy and cardiac assessment.

Our observations of biomarker fluctuations following Delandistrogene Moxeparvovec (Elevidys) infusion align with safety patterns reported in clinical trials.

### Age and biomarker trends

As the initial approval for gene therapy was limited to patients below 6 years of age, we divided our cohort into those ≤6 years and >6 years for descriptive purposes. While we observed some differences in the pattern of biomarker fluctuations (muscle enzymes, troponin, and liver function markers). Younger patients (≤6 years) appeared to have greater variability in AST, ALT, and CK levels in the early weeks post-infusion, while older patients showed transient elevations in Troponin I and a gradual rise in GGT.

These trends may reflect differences in underlying disease stage or organ vulnerability at the time of treatment. Fluctuations in the younger aged patients may indicate increased muscle turnover, inflammation, or muscle repair in response to the gene therapy.

Older patients showed transient peaks in Troponin I levels at weeks 1 and 3, which may suggest temporary cardiac stress post-infusion. Younger patients in our series maintained consistently low Troponin I levels, with no clear evidence of cardiac impact observed. These preliminary observations highlight the importance of careful cardiac monitoring in all patients receiving gene therapy, regardless of age. Transient troponin I elevations in older patients at Weeks 1 and 3 mirrored the cardiac safety signals described in early Delandistrogene Moxeparvovec (Elevidys) trials, in which myocarditis and troponin rises were documented, thereby supporting ongoing recommendations for routine cardiac monitoring during the first month post-treatment (6 & 9). By contrast, consistently low troponin I levels in our younger cohort may indicate greater cardiac resilience at earlier disease stages in our cohort, younger children (≤6 years) exhibited early variability in AST and ALT, consistent with reports of treatment-associated transaminitis and acute liver enzyme elevations typically occurring in the first 8 weeks post-infusion in phase I/II and phase III programs, which necessitated weekly hepatic monitoring in this period [8]. Conversely, older patients demonstrated a more gradual increase in GGT from Week 4 onward, suggesting that age may modulate hepatic response, although larger studies are required to validate this observation.

Clinical trial studies have primarily emphasized molecular and functional endpoints, reporting robust micro-dystrophin expression (34–51% of normal by Western blot at 12 weeks, with some cohorts reaching >90% expression) alongside early functional trends in NSAA scores and timed function tests (7 & 10). Restoration of dystrophin is expected to stabilize the sarcolemma, thereby reducing membrane fragility during muscle contraction. This structural improvement is often reflected biochemically as a reduction in serum creatine kinase (CK) levels, which are widely used as surrogate markers of muscle fiber breakdown [6]. In our series, the variability in CK trends—particularly among younger patients—may reflect the interplay between active muscle regeneration and dystrophin restoration. Although CK reductions have been documented in clinical trials following successful gene transfer, the interpretation of CK kinetics in real-world cohorts requires caution, given age-related differences in muscle mass, physical activity, and ongoing degeneration/regeneration cycles.

Taken together, these findings reinforce the importance of integrating serial hepatic and cardiac biomarkers into post-infusion monitoring protocols. Future prospective studies should combine biochemical, molecular, and functional endpoints to better delineate age-related trajectories and long-term outcomes following gene therapy in Duchenne muscular dystrophy.

### Timed efficacy assessment

In our cohort of eight DMD patients who received Delandistrogene Moxeparvovec (Elevidys), functional assessments (NSAA, 10MWT, and get-up time) were performed prior to gene therapy, and then at one and two months post-infusion. Two of the eight patients were non-ambulatory, rendering NSAA an invalid outcome measure for this subgroup. Additionally, one international patient returned home after infusion, and one was lost to follow-up, leaving only four patients with valuable serial assessments. Among these, two demonstrated improvement, one remained unchanged, and one showed a decline in NSAA over two months. Importantly, the NSAA decline coincided with a 3.5 kg weight gain, likely steroid-related, while this patient continued to improve in both 10MWT and get-up time, suggesting that the observed NSAA decline may not reflect a true functional deterioration.

Our results highlight several limitations of NSAA as a sole functional outcome measure. For example, Patient 2 had no change in NSAA at two months but demonstrated measurable gains in 10MWT and get-up time, with further improvement in NSAA only observed at the four-month reassessment. These findings underscore the importance of complementary functional metrics and extended follow-up to fully capture therapeutic benefit in DMD gene therapy.

When compared with published Elevidys trials, our observations are aligned with key themes. In a 2020 study by Mendell et al. [8], small cohorts of ambulatory boys (*n* = 4 per dose group) demonstrated improvements in NSAA from baseline at 48 weeks, but variability between individuals was significant. Similarly, in EMBARK [9, 10], although the primary endpoint of NSAA change at 52 weeks did not reach statistical significance across the cohort, subsets of patients showed clinically meaningful improvements in NSAA and timed function tests. These trial results align with our experience: functional gains were heterogeneous, and short-term outcomes could miss delayed improvements that emerged later. Moreover, as in our cohort, weight gain and corticosteroid exposure have been recognized as potential confounders of functional scores in Elevidys studies.

The primary limitations of our study are the small sample size (eight patients, with only four evaluable on serial testing) and the short duration of follow-up. These constraints parallel those in early Delandistrogene Moxeparvovec (Elevidys) trials, where interpretation of results was limited by low patient numbers and relatively short observation periods. Longer-term follow-up with broader functional outcome measures is essential to confirm and contextualize early post-gene-therapy changes.

### Caregiver feedback

All parents reported positive functional changes within two months of receiving gene therapy, though four out of five expressed concerns about weight gain following treatment. One patient remained non-ambulatory and could not be timed for the 10MWT or perform a floor rise, while among the others, one demonstrated progressive improvement in the 10MWT speed over two months, two showed a decline, and one remained unchanged. These results, taken together with parent observations, suggest that steroid-induced weight gain may be affecting patient function post gene therapy.

### Effects of prednisolone and supportive protocols

The use of prednisolone at 2 mg/kg/day started 2 days pre-infusion, tapered to a maintenance dose over three months, likely contributed to the favorable biomarker trends observed across both age groups. Prednisolone helped manage immune responses, minimizing severe inflammatory reactions. The administration of pre-infusion IV methylprednisolone, diphenhydramine, and ondansetron further supported patient safety by reducing the risk of allergic reactions, nausea, and other infusion-related side effects.

The structured monitoring protocol, including ICU-based infusions and weekly follow-up clinic visits, was essential in detecting and managing side effects. The use of regular physiotherapy likely supported muscle function, contributing to the observed stabilization in CK levels post-therapy. The overall tolerability was high, with only mild side effects like corticosteroid-induced weight gain observed in most patients.

Despite clinical trials showing that after infusion with Delandistrogene Moxeparvovec (Elevidys) severe myocarditis and elevated Troponin I levels may occur [11], none of our patients exhibited signs of myocarditis, as confirmed by clinical assessments, laboratory results, electrocardiograms, and echocardiographic findings after one month of treatment. Although this is a good indicator of the short-term safety of the medication, long-term follow-up is indicated to assess the safety of the medicine

### Clinical implications

The distinct biomarker trends observed between age groups emphasize the importance of age-specific management strategies in gene therapy for DMD: The stable biomarker responses in younger versus older patients indicate that early gene therapy intervention, paired with supportive care, may be more effective in stabilizing muscle and cardiac health, potentially slowing disease progression with minimal adverse effects.

## Conclusion

This study demonstrates that Delandistrogene Moxeparvovec (Elevidys) is well tolerated in DMD patients aged 4–11 years. The findings indicate the safety and efficacy of gene therapy in DMD when combined with a protocol that includes antibody screening, tailored corticosteroid use, physiotherapy, and regular cardiac and hepatic monitoring. Further larger real-world data is required to further confirm the safety and efficacy of Delandistrogene moxeparvovec for treatment of DMD patients.

## Supplementary information

Supplemental table 1

Supplemental table 2

Supplemental table 3

Supplemental table 4

Supplemental table 5

Supplemental table 6

Supplemental table 7

Supplemental table 8

Supplementary Figure 1

## Data Availability

The original contributions presented in this study are included in the article, further inquiries can be directed to the corresponding author.
