# Supplementary material for: Real-world experience with gene therapy in Duchenne muscular dystrophy center readiness and patients safety: report from Qatar
Source: Gene Ther. 2025 Nov 27;33(1):78–83. doi: 10.1038/s41434-025-00580-3 (PMC12932109; doi:10.1038/s41434-025-00580-3)
Supplement: Supplementary file 1 — Supplemental table 1 [file 41434_2025_580_MOESM1_ESM.docx]

**Supplementary table 1.**

*Patients AST levels 30 weeks post gene therapy. AST: 23-46 IU/L*

| **Patient** | **Pre-Infusion** | **Week 1 Post Infusion** | **Week 2** | **Week 3** | **Week 4** | **Week 5** | **Week 6** | **Week 7** | **Week 8** | **Week 10** | **Week 14** | **Week 18** | **Week 22** | **Week 26** | **Week 30** |
| --- | --- | --- | --- | --- | --- | --- | --- | --- | --- | --- | --- | --- | --- | --- | --- |
| 1 | 511 | 783 | 274 | 230 | 227 | 302 | 256 | 192 | 151 | 116 | 169 | - | - | - | - |
| 2 | 52 | 32 | 22 | 21 | 19 | 18 | 20 | 28 | 140 | 215 | 72 | 89 | 74 | 74 | 54 |
| 3 | 53 | 38 | 59 | - | 30 | 28 | 22 | 25 | 24 | 38 | 313 | 116 | 81 | 47 | 33 |
| 4 | 306 | 137 | 108 | 238 | 77 | 140 | 279 | 78 | 50 | 78 | 150 | 93 | 177 | 480 | 252 |
| 5 | 453 | 81 | 54 | 60 | 42 | 38 | 53 | 42 | 39 | 43 | 59 | 71 | 92 | 93 | 112 |
| 6 | 87 | 128 | 113 | 92 | 62 | 97 | - | 50 | 81 | 125 | 69 | - | 146 | 192 | 145 |
| 7 | 141 | 39 | - | 24 | - | 28 | - | 98 | 70 | - | 52 | - | - | - | 79 |
| 8 | 222 | 102 | 786 | 261 | 184 | 176 | 135 | 222 | 97 | 140 | 207 | - | - | - | - |
