# Supplementary material for: Real-world experience with gene therapy in Duchenne muscular dystrophy center readiness and patients safety: report from Qatar
Source: Gene Ther. 2025 Nov 27;33(1):78–83. doi: 10.1038/s41434-025-00580-3 (PMC12932109; doi:10.1038/s41434-025-00580-3)
Supplement: Supplementary file 2 — Supplemental table 2 [file 41434_2025_580_MOESM2_ESM.docx]

**Supplementary table 2.**

*Patients ALT levels 30 weeks post gene therapy. ALT: 10-25 IU/L*

| **Patient** | **Pre-Infusion** | **Week 1 Post Infusion** | **Week 2** | **Week 3** | **Week 4** | **Week 5** | **Week 6** | **Week 7** | **Week 8** | **Week 10** | **Week 14** | **Week 18** | **Week 22** | **Week 26** | **Week 30** |
| --- | --- | --- | --- | --- | --- | --- | --- | --- | --- | --- | --- | --- | --- | --- | --- |
| 1 | 389 | 338 | 324 | 248 | 245 | 264 | 237 | 167 | 130 | 114 | 161 | - | - | - | 86 |
| 2 | 65 | 56 | 41 | 37 | 34 | 34 | 37 | 38 | 176 | 183 | 107 | 86 | 80 | 81 | 65 |
| 3 | 104 | 100 | 129 | - | 83 | 86 | 66 | 65 | 60 | 75 | 400 | 269 | 187 | 79 | 49 |
| 4 | 395 | 229 | 358 | 395 | 213 | 241 | 332 | 228 | 109 | 152 | 306 | 226 | 365 | 517 | 473 |
| 5 | 198 | 203 | 160 | 135 | 107 | 86 | 91 | 89 | 76 | 76 | 92 | 110 | 115 | 122 | 119 |
| 6 | 142 | 184 | 192 | 205 | 144 | 120 | - | 113 | 144 | 242 | 141 | - | 201 | 183 | 184 |
| 7 | 139 | 32 | 37 | 32 | 35 | 46 | - | 126 | 93 | - | 66 | - | - | - | 83 |
| 8 | 353 | 197 | 433 | 450 | 337 | 298 | 245 | 229 | 188 | 189 | 227 | - | - | - | - |
