# Supplementary material for: Real-world experience with gene therapy in Duchenne muscular dystrophy center readiness and patients safety: report from Qatar
Source: Gene Ther. 2025 Nov 27;33(1):78–83. doi: 10.1038/s41434-025-00580-3 (PMC12932109; doi:10.1038/s41434-025-00580-3)
Supplement: Supplementary file 3 — Supplemental table 3 [file 41434_2025_580_MOESM3_ESM.docx]

**Supplementary table 3.**

*Patients CK levels 30 weeks post gene therapy. CK: 45-302 IU/L*

| **Patient** | **Pre-Infusion** | **Week 1 Post Infusion** | **Week 2** | **Week 3** | **Week 4** | **Week 5** | **Week 6** | **Week 7** | **Week 8** | **Week 10** | **Week 14** | **Week 18** | **Week 22** | **Week 26** | **Week 30** |
| --- | --- | --- | --- | --- | --- | --- | --- | --- | --- | --- | --- | --- | --- | --- | --- |
| 1 | 16,694 | 24,964 | 6,724 | 5,640 | 4,964 | 7,163 | 6,965 | 6,064 | 4,357 | 2,364 | 4,662 | - | - | - | - |
| 2 | 2,405 | 100 | 79 | 91 | 46 | 52 | 40 | 80 | 391 | 202 | 81 | 411 | 494 | 279 | 213 |
| 3 | 3,565 | 1,115 | 1,496 | - | 436 | 213 | 177 | 226 | 239 | 466 | 280 | 774 | 567 | 838 | 444 |
| 4 | 15,395 | 5,831 | 4,735 | 8,755 | 1,984 | 4,931 | 8,975 | 1,676 | 919 | 1,661 | 3,264 | 2,554 | 5,247 | 5,993 | 7,203 |
| 5 | 8,093 | 2,695 | 1,286 | 1,931 | 845 | 823 | 1,334 | 1,139 | 792 | 1,095 | 1,133 | 1,601 | 2,317 | 3,276 | 4,076 |
| 6 | 3,816 | 4,803 | 3,772 | 2,648 | 1,059 | 2,876 | - | 1,175 | 1,422 | 1,981 | 1,064 | - | 2,990 | 6,964 | 4,599 |
| 7 | 5,065 | 480 | 377 | 194 | 218 | 174 | - | - | 1,222 | - | 593 | - | - | - | 2,922 |
| 8 | 8,790 | 3,017 | - | 6,026 | 4,720 | 3,847 | 2,371 | 7,413 | 1,567 | 3,690 | 4,639 | - | - | - | - |
