# Supplementary material for: Real-world experience with gene therapy in Duchenne muscular dystrophy center readiness and patients safety: report from Qatar
Source: Gene Ther. 2025 Nov 27;33(1):78–83. doi: 10.1038/s41434-025-00580-3 (PMC12932109; doi:10.1038/s41434-025-00580-3)
Supplement: Supplementary file 4 — Supplemental table 4 [file 41434_2025_580_MOESM4_ESM.docx]

**Supplementary table 4.**

*Patients GGT levels 30 weeks post gene therapy. GGT: 5-14 IU/L*

| **Patient** | **Pre-Infusion** | **Week 1 Post Infusion** | **Week 2** | **Week 3** | **Week 4** | **Week 5** | **Week 6** | **Week 7** | **Week 8** | **Week 10** | **Week 14** | **Week 18** | **Week 22** | **Week 26** | **Week 30** |
| --- | --- | --- | --- | --- | --- | --- | --- | --- | --- | --- | --- | --- | --- | --- | --- |
| 1 | 7 | 9 | 10 | 11 | 11 | 9 | 10 | 11 | 10 | 10 | 8 | - | - | - | 70 |
| 2 | 20 | 25 | 26 | 28 | 32 | - | 31 | 27 | 30 | 28 | 23 | 17 | 18 | 22 | - |
| 3 | 16 | 20 | 19 | - | 28 | 35 | 29 | 26 | - | 30 | 100 | 120 | 91 | 38 | 26 |
| 4 | 5 | 10 | 9 | 12 | 14 | 14 | 14 | 17 | 18 | 17 | 13 | 17 | 18 | 18 | 11 |
| 5 | 25 | 27 | 27 | 31 | 34 | 33 | 28 | 30 | 29 | - | 37 | 40 | 25 | - | - |
| 6 | 14 | 15 | 15 | 18 | 20 | 18 | - | 16 | 18 | 20 | 17 | - | 15 | 17 | 16 |
| 7 | 14 | 22 | - | - | - | - | - | 46 | - | - | - | - | - | - | - |
| 8 | 8 | 12 | - | 15 | 15 | 17 | 17 | 18 | 21 | 20 | 21 | - | - | - | - |
