# Supplementary material for: Real-world experience with gene therapy in Duchenne muscular dystrophy center readiness and patients safety: report from Qatar
Source: Gene Ther. 2025 Nov 27;33(1):78–83. doi: 10.1038/s41434-025-00580-3 (PMC12932109; doi:10.1038/s41434-025-00580-3)
Supplement: Supplementary file 5 — Supplemental table 5 [file 41434_2025_580_MOESM5_ESM.docx]

**Supplementary table 5.**

*Patients Troponin-I levels 30 weeks post gene therapy.* *Troponin I: 0-20 ng/L*

| **Patient** | **Pre-Infusion** | **Week 1 Post Infusion** | **Week 2** | **Week 3** | **Week 4** | **Week 5** | **Week 6** | **Week 7** | **Week 8** | **Week 10** | **Week 14** | **Week 18** | **Week 22** | **Week 26** | **Week 30** |
| --- | --- | --- | --- | --- | --- | --- | --- | --- | --- | --- | --- | --- | --- | --- | --- |
| 1 | 6 | 9 | 7 | 8 | 11 | 16 | 12 | 9 | 10 | 7 | 9 | - | - | - | - |
| 2 | 4 | 5 | 5 | 6 | 8 | 6 | 4 | 6 | 8 | 5 | 5 | 6 | 5 | 3 | - |
| 3 | 51 | 34 | 34 | - | 45 | 33 | 37 | 30 | 22 | - | 26 | 23 | 30 | 44 | 24 |
| 4 | 8 | 5 | 4 | 5 | 5 | 5 | 4 | 4 | 5 | 4 | 5 | 12 | 8 | 8 | 9 |
| 5 | 3 | 4 | - | 6 | 5 | 5 | 6 | 6 | 12 | 6 | 7 | 11 | 8 | - | 11 |
| 6 | 8 | 10 | 11 | 9 | 7 | 10 | 8 | - | 8 | 8 | 8 | - | 7 | 7 | 8 |
| 7 | 3 | 3 | - | 10 | - | 10 | - | - | - | - | 10 | - | - | - | - |
| 8 | 5 | 3 | - | 11 | 9 | 13 | 8 | 12 | 11 | 8 | 8 | - | - | - | - |
