# Supplementary material for: Real-world experience with gene therapy in Duchenne muscular dystrophy center readiness and patients safety: report from Qatar
Source: Gene Ther. 2025 Nov 27;33(1):78–83. doi: 10.1038/s41434-025-00580-3 (PMC12932109; doi:10.1038/s41434-025-00580-3)
Supplement: Supplementary file 6 — Supplemental table 6 [file 41434_2025_580_MOESM6_ESM.docx]

**Supplementary table 6.**

*Patients LDH levels 30 weeks post gene therapy. LDH 200-333 IU/L*

| **Patient** | **Pre-Infusion** | **Week 1 Post Infusion** | **Week 2** | **Week 3** | **Week 4** | **Week 5** | **Week 6** | **Week 7** | **Week 8** | **Week 10** | **Week 14** | **Week 18** | **Week 22** | **Week 26** | **Week 30** |
| --- | --- | --- | --- | --- | --- | --- | --- | --- | --- | --- | --- | --- | --- | --- | --- |
| 1 | 772 | 1537 | 806 | - | - | - | - | 628 | - | - | 462 | - | - | - | - |
| 2 | 340 | 371 | 419 | 471 | 506 | - | 747 | 719 | 675 | 568 | 393 | 399 | 327 | 390 | - |
| 3 | 421 | 376 | 618 | - | 482 | 597 | 547 | 549 | 548 | - | - | 462 | 386 | 465 | 287 |
| 4 | 1609 | 901 | 1170 | 1506 | 729 | 870 | 1120 | 756 | 492 | 639 | 725 | 674 | 1039 | 900 | 769 |
| 5 | 602 | 542 | 511 | 567 | 557 | 566 | - | - | - | - | - | - | 429 | - | - |
| 6 | 365 | 503 | 498 | 505 | 440 | 509 | - | - | - | - | 344 | - | 378 | - | 396 |
| 7 | 483 | 356 | - | - | - | - | - | - | - | - | - | - | - | - | 286 |
| 8 | 1130 | 620 | - | 1175 | 878 | - | 615 | 801 | 594 | 622 | 722 | - | - | - | - |
