# Supplementary material for: Real-world experience with gene therapy in Duchenne muscular dystrophy center readiness and patients safety: report from Qatar
Source: Gene Ther. 2025 Nov 27;33(1):78–83. doi: 10.1038/s41434-025-00580-3 (PMC12932109; doi:10.1038/s41434-025-00580-3)
Supplement: Supplementary file 7 — Supplemental table 7 [file 41434_2025_580_MOESM7_ESM.docx]

**Supplementary table 7.**

*Patients Platelets levels 30 weeks post gene therapy*. *Platelets: 150-400/L*

| **Patient** | **Pre-Infusion** | **Week 1 Post Infusion** | **Week 2** | **Week 3** | **Week 4** | **Week 5** | **Week 6** | **Week 7** | **Week 8** | **Week 10** | **Week 14** | **Week 18** | **Week 22** | **Week 26** | **Week 30** |
| --- | --- | --- | --- | --- | --- | --- | --- | --- | --- | --- | --- | --- | --- | --- | --- |
| 1 | 346 | 296 | 294 | 364 | 305 | 312 | 398 | 400 | 360 | 347 | 350 | - | - | - | 133 |
| 2 | 500 | 574 | 393 | 408 | 347 | 336 | 343 | 374 | 294 | 354 | 286 | 398 | 355 | 343 | - |
| 3 | 373 | 439 | 314 | - | 343 | 247 | 255 | 275 | 310 | - | 368 | 287 | 316 | 369 | 347 |
| 4 | 451 | 477 | 236 | 649 | 587 | 520 | 461 | 563 | 507 | 508 | 567 | 500 | 457 | 455 | 500 |
| 5 | 391 | 360 | 417 | 416 | 380 | 335 | 346 | 348 | 387 | 340 | 305 | 359 | 354 | 349 | 407 |
| 6 | 406 | 359 | 349 | 330 | 323 | 327 | - | 290 | 330 | 259 | 324 | - | 311 | 349 | 418 |
| 7 | 419 | 259 | - | 422 | 312 | 392 | - | 284 | 378 | - | 387 | - | - | - | 562 |
| 8 | 574 | 520 | 451 | 492 | 453 | 398 | 359 | 459 | 446 | 430 | 343 | - | - | - | - |
