# Supplementary material for: Real-world experience with gene therapy in Duchenne muscular dystrophy center readiness and patients safety: report from Qatar
Source: Gene Ther. 2025 Nov 27;33(1):78–83. doi: 10.1038/s41434-025-00580-3 (PMC12932109; doi:10.1038/s41434-025-00580-3)
Supplement: Supplementary file 8 — Supplemental table 8 [file 41434_2025_580_MOESM8_ESM.docx]

**Supplementary table 8.**

*Patients INR levels 30 weeks post gene therapy.* *INR: 0.8-1.2*

| **Patient** | **Pre-Infusion** | **Week 1 Post Infusion** | **Week 2** | **Week 3** | **Week 4** | **Week 5** | **Week 6** | **Week 7** | **Week 8** | **Week 10** | **Week 14** | **Week 18** | **Week 22** | **Week 26** | **Week 30** |
| --- | --- | --- | --- | --- | --- | --- | --- | --- | --- | --- | --- | --- | --- | --- | --- |
| 1 | 1.1 | 0.9 | 0.9 | - | - | - | - | 1.0 | - | - | 0.9 | - | - | - | - |
| 2 | 1.1 | 1.0 | 0.9 | 0.9 | 0.9 | 0.9 | 0.9 | 0.9 | 1.0 | 1.0 | 0.9 | 1.0 | 1.0 | 1.0 | - |
| 3 | 1.0 | 0.9 | 0.9 | - | 0.9 | 0.9 | - | - | - | - | - | - | - | - | - |
| 4 | 1.1 | 1.0 | 1.0 | 1.0 | 1.0 | 1.0 | 1.0 | 1.0 | 1.0 | 1.0 | 1.0 | 1.0 | 1.0 | 1.0 | 1.1 |
| 5 | 1.1 | 1.0 | 1.0 | 1.0 | 1.0 | 1.0 | - | - | - | - | 1.1 | - | 1.0 | - | - |
| 6 | 0.9 | 0.8 | 0.8 | 0.8 | 0.8 | 0.8 | - | - | - | - | - | - | 0.9 | 0.9 | 0.9 |
| 7 | 1.1 | 1.0 | - | - | - |  | - | - | - | - | 1.1 | - | - | - | 1.1 |
| 8 | 1.0 | 1.1 | - | 0.9 | 0.9 | 0.9 | 0.9 | 0.9 | 0.9 | 0.9 | 0.9 | - | - | - | - |
