# Supplementary material for: Real-world experience with gene therapy in Duchenne muscular dystrophy center readiness and patients safety: report from Qatar
Source: Gene Ther. 2025 Nov 27;33(1):78–83. doi: 10.1038/s41434-025-00580-3 (PMC12932109; doi:10.1038/s41434-025-00580-3)
Supplement: Supplementary file 9 — Supplementary Figure 1 [file 41434_2025_580_MOESM9_ESM.docx]

**Supplementary Figure 1.**

*CK and Troponin-1 levels of patient 3.*
